# Species Delimitation Methods Facilitate the Identification of Cryptic Species Within the Broadly Distributed Species in *Homoeocerus* (*Tliponius*) (Insecta: Hemiptera: Coreidae) †

**DOI:** 10.3390/insects16080797

**Published:** 2025-08-01

**Authors:** Jingyu Liang, Shujing Wang, Jingyao Zhang, Juhong Chen, Siying Fu, Zhen Ye, Huai-Jun Xue, Yanfei Li, Wenjun Bu

**Affiliations:** Institute of Entomology, College of Life Sciences, Nankai University, Tianjin 300071, China; jljy47ky@163.com (J.L.); wangsj@nankai.edu.cn (S.W.); 2120231463@mail.nankai.edu.cn (J.Z.); 15181674153@163.com (J.C.); nkufsy@163.com (S.F.); yezhen1987331@nankai.edu.cn (Z.Y.); xuehj@nankai.edu.cn (H.-J.X.)

**Keywords:** *Tliponius*, species delimitation, mitogenome, phylogeny, *Homoeocerus*

## Abstract

Widespread species may exhibit considerable genetic variation among populations due to their extensive distribution ranges, and may even give rise to new species in remote areas. Integrative species delimitation via multiple types can provide a robust framework for accurate species identification and rapid discovery of cryptic diversity. The subgenus *Tliponius* (Hemiptera: Coreidae: *Homoeocerus*) has several species and three broadly distributed species across China. In this study, we selected as many geographical sample sites of widely distributed species as possible and conducted species identification based on integrated taxonomy of morphological, mitochondrial and SNP data for 28 individuals within *Tliponius.* Our results revealed a cryptic lineage previously subsumed under the polytypic *H. unipunctatus* in Yunnan Province and described as *Homoeocerus (Tliponius) dianensis* Liang, Li & Bu sp. nov. The presence of seven distinct species within *Tliponius* was supported by species delimitation and divided into two clades: (*H. dilatatus* + (*H. marginellus* + (*H. unipunctatus + H. dianensis* sp. nov.))) and (*H. yunnanensis* + (*H. laevilineus* + *H. marginiventris*). Based on our findings, extensive sampling of widespread species is highly important for the accuracy of species delimitation and the discovery of cryptic species.

## Simple Summary

Integrative species delimitation based on multiple types of data, including morphology and molecular biology, can accurately identify species and rapidly detect cryptic species. Comprehensive sampling is crucial for the accuracy of species delimitation in widely distributed species. In this study, based on extensive geographical sampling and multitype data, species delimitation and population genetics methods were used to clarify the species boundaries within the widely distributed species in the subgenus *Tliponius* in China, and a cryptic species from Yunnan Province was identified and named *Homoeocerus* (*Tliponius*) *dianensis* Liang, Li & Bu sp. nov. This study provides insights for species delimitation research based on extensive sampling and multitype data involving widely distributed species.

## 1. Introduction

Species delimitation is a fundamental component of systematic biology, providing the framework for understanding the evolutionary processes that generate biodiversity [1]. In recent years, with the diversification of molecular data acquisition methods and the popularization of species delimitation approaches, species discovery has accelerated significantly [2,3]. Currently, multi-lineage evidence-based species delimitation methods have been widely applied in taxonomic studies across diverse clades [4]. By integrating multi-lineage evidence (morphology, mitochondrial genes, nuclear genes and etc.) and multiple analytical approaches (phylogenetics, genetic distance analysis, population genetics), cryptic species and closely related species can be effectively identified [5,6]. Accurate species identification provides a precise scientific basis for defining species boundaries and refining taxonomic systems. The integration and mutual corroboration of data have gradually become key evidence for species discovery, particularly through the mutual validation of morphological and molecular data [7]. Closely related species often exhibit highly similar morphologies that are difficult to distinguish, whereas molecular data have enabled the rapid discovery and identification of independent genetic lineages [8].

As widely distributed species often span multiple ecological regions due to their extensive geographic ranges, significant genetic and phenotypic differentiation frequently occurs within populations as a result of geographic isolation, environmental heterogeneity, or local adaptation [9]. In recent years, with the advancement of molecular systematics and population genetics, an increasing number of widely distributed species previously regarded as single taxonomic units have been found to contain multiple cryptic species [2]. This phenomenon has been widely reported across plant, animal, and microbial lineages, indicating that traditional morphological species may severely underestimate biodiversity [10,11,12,13]. In particular, in regions with complex geographic topography and high environmental heterogeneity, widely distributed species are more prone to genetic divergence due to geographic isolation or ecological adaptation, thereby forming more cryptic species [14]. For widely distributed species, intraspecific genetic differentiation often occurs, and cases where genetic distances are greater between marginal populations or geographically distant populations exist. However, whether such differentiation reaches the species level requires the comprehensive integration of multiple lines of evidence. Additionally, the comprehensiveness of taxon sampling is highly recommended to resolve the issue. Based on extensive sampling, comprehensive analyses incorporating multiple datasets (e.g., morphology, mitochondrial genes, and nuclear genes) are more persuasive for determining the explicit taxonomic status of species [15,16,17].

The true bug subgenus *Tliponius* Stål, classified under the genus *Homoeocerus*, is characterized by a yellowish-brown, elliptical to fusiform body, a quadrate head, and a broad, short apicolateral angle of the corium; some species bears a central black spot on the corium. Currently, the 44 recognized species of *Tliponius* share relatively similar morphological traits and are mainly distributed in tropical and temperate Asia within the Oriental region. Among the eleven species from China, three species are widespread, even in East Asia, whereas the other species have relatively narrowly distributions [18]. The three widespread species, *H.* (*T.*) *dilatatus* Horvath, *H.* (*T.*) *marginellus* (Herrich-Schaeffer) and *H.* (*T.*) *unipunctatus* (Thunberg), are recorded as the pests of soybeans and other crops [19]. In preliminary study, *H. unipunctatus* (Thunberg) had some synonyms in its taxonomic history [20]. Hsiao (1962) found some specimens with certain color pattern variations in antennae in Yunnan that appeared to correspond to *H. distinctus* described by Signoret [21]. However, he ultimately regarded them as a variety of *H. unipunctatus* and treated the name as a junior synonym [22]. Previous research on *Tliponius* has focused predominantly on morphological descriptions, with very limited molecular data available. Only mitochondrial sequences from partial species within the genus *Homoeocerus* have been documented, and no comprehensive species delimitation or phylogenetic studies based on full sampling have been conducted for this subgenus.

In this study, we integrated morphological data, mitochondrial sequences, and simplified nuclear genome data (ddRAD-Seq), combined with species delimitation methods, to: (a) assess the clarity of species boundaries within *Tliponius* in China, especially for the widespread species; (b) determine whether the population in *H. unipunctatus* from Yunnan province represented a cryptic species and (c) evaluate the phylogenetic relationships among species of *Tliponius* in China.

## 2. Materials and Methods

### 2.1. Sample Acquisition and DNA Extraction

**Figure 1:**
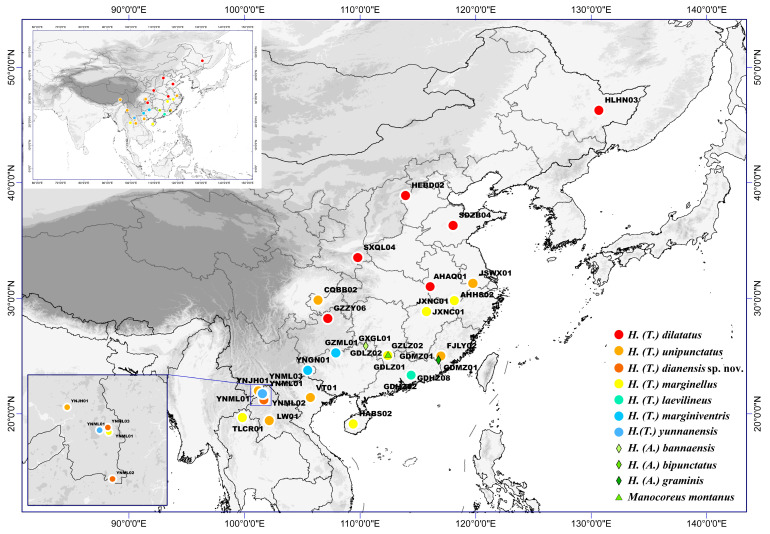
Geographical map of all sampling sites.

Our study included 28 samples of the subgenus *Tliponius* in *Homoeocerus* from China and the Indochina Peninsula (Figure 1; Table S1). The specimens were preliminarily identified and classified based on original descriptions [22]. Six species of *Tliponius* (including *Homoeocerus dilatatus*, *H. unipunctatus*, *H. marginellus*, *H. laevilineus*, *H. marginiventris*, and *H. yunnanensis*) were identified. After genetic analyses, morphological analysis was carried out to confirm the species classification based on molecular data. Since *H. dilatatus*, *H. unipunctatus*, and *H. marginellus* are relatively widespread, we selected multiple samples from different locations to cover their distribution areas. We chose 3 species from another subgenus *Anacanthocoris* in *Homoeocerus* in China and one species in other genus, *Manocoreus montanus*, as outgroup taxa.

All samples collected were immediately preserved in 100% ethanol in the wild and stored in a freezer at −20 °C before DNA extraction. Genomic DNA was extracted from the thoracic muscle using a Universal Genomic DNA Kit (CWBIO, Beijing, China). The DNA and sample vouchers were deposited at the College of Life Sciences at Nankai University, Tianjin, China (NKU).

### 2.2. Morphological Identification

Specimens were examined and measured with an Olympus SZX7 stereomicroscope (Olympus Corporation, Tokyo, Japan). General habitus images were captured using a Canon EOS 5D Mark II DSLR (Canon Inc., Tokyo, Japan) fitted with a Laowa 60 mm f/2.8 2× macro lens (Venus Optics, Hefei, China). Focus stacks were generated with Helicon Focus v7.6.1 (Helicon Soft, Kharkiv, Ukraine; https://www.heliconsoft.com). For detailed imaging of the male genitalia, genital segments were cleared in warm 10% KOH to dissolve soft tissues, then photographed using a 10× objective lens on an OLYMPUS BX53F microscope (Olympus Corporation, Tokyo, Japan) equipped with an OLYMPUS DP72 digital camera (Olympus Corporation, Tokyo, Japan). All measurements are reported in millimeters (mm).

### 2.3. Molecular Data Acquisition

#### 2.3.1. Mitochondrial Datasets and Sequence Analyses

We sequenced the mitogenomes of 32 samples (including the four outgroups). Sequencing was performed by Novogene (Beijing, China) using the Illumina NovaSeq 6000 platform (Illumina, San Diego, CA, USA) to obtain 150 bp paired-end reads.

The mitogenomes were assembled in two strategies for assembly and mutual verification. De novo assembly was performed by using MitoZ v1.03 [23] and IDBA-master [24]. Mapping to reference genomes was conducted by MITObim v1.9 [25] and Geneious v2020.2.1 [26], which took *Homoeocerus unipunctatus* as the reference genome (GenBank accession no. MW619675).

The Mitos Web Server (original version: http://mitos.bioinf.uni-leipzig.de/, accessed on 6 February 2023; server now moved to: https://usegalaxy.eu/ and search for "MITOS") was used to identify the boundaries of tRNA genes and the secondary structures of tRNAs [27]. The start and stop codons of protein-coding genes (PCGs) are determined by ORF Finder via invertebrate mitochondrial genetic codes, which are implemented by the NCBI website (https://www.ncbi.nlm.nih.gov/orffinder/, accessed on 6 February 2023) and the boundaries of PCGs and rRNA genes are confirmed by an alignment with homologous genes from published heteropteran mitogenomes. Two rRNA boundaries are predicted by comparison with homologous regions of the published mitogenome of MW619675.

One mitochondrial dataset of 32 samples was obtained. Amino acid alignments of 13 PCGs were performed using the MUSCLE algorithm in MEGA X [28]. The genes for each sample were concatenated into a combined matrix (32PCGs), and all three codon positions of the 13 PCGs were used in subsequent analyses. MEGA X was used to calculate the nucleotide composition of the 13 PCGs. The GC content in the 13 PCGs of *Tliponius* was analyzed by analysis of variance (ANOVA) in the R package agricolae. In addition, a COI dataset of 32 samples (32COI) was also extracted for subsequent analyses.

#### 2.3.2. ddRAD-Seq Library Preparation, Sequencing, and Data Processing

The ddRAD-seq library was prepared based on all 32 samples following a protocol provided in a previous study [29]. 300 ng DNA of each sample was used for double digestion with *Eco*RI and *Msp*I restriction enzymes (New England Biolabs, Ipswich, MA, USA). Illumina adapters and unique 5-bp barcodes were then ligated to the two ends of the digested fragments. After ligation, the products were pooled together (30 samples with unique 5-bp barcodes formed one ddRAD library), followed by amplification of size-selected pools with 8–10 PCR cycles using Illumina-indexed primers. The PCR products were pooled and run through a Pippin Prep instrument (Sage Science, Beverly, MA, USA) to select fragments from 300 to 600 bp; the size-selected pools were then amplified with 8–10 PCR cycles using Illumina-indexed primers. The enzyme-digested products, ligation products, and amplified DNA fragments from each sample were purified using AMPure XP magnetic beads (Beckman Coulter Inc., Brea, CA, USA). Finally, 150-bp paired-end reads were generated on an Illumina HiSeq X10 platform at the Novogene Sequencing Center & CAP and ISO Lab.

The raw Illumina reads were processed with the ‘API: ipyrad assembly workflow’ [30]. First, each individual was separated into independent read files. Then, the number of reads per individual was confirmed and performed in subsequent analysis. The parameter settings for the ipyrad assembly were as follows: the filter for the adapters was set to 2, the clustering threshold for de novo assembly was set to 0.85, and all other parameters were set to default values.

Two single-nucleotide polymorphisms (SNP) datasets were constructed for population genetic analysis. The SNP dataset based on 32 samples (32SNP) was constructed with 40% missing data. We also generated two subset datasets with only 28 samples in *Tliponius* (28SNP) to delimit related species. To avoid linkage across sites within the same locus, one random SNP was sampled from each locus and an uSNPs dataset (28uSNP) was also generated for downstream analyses.

### 2.4. Phylogenetic Inference and Divergence Time Estimation

The phylogenetic analyses were conducted utilizing Bayesian inference (BI) and maximum likelihood (ML). PartitionFinder2 was used to determine the optimal partitioning strategies and the best-fit nucleotide substitution model [31]. The results of PartitionFinder2 for MrBayes v3.2.5 [32] and IQ-TREE v2 [33] revealed the best-fit nucleotide substitution model. BI analyses were performed by MrBayes 3.2.5 with Markov chain Monte Carlo analysis (four chains) run for 10,000,000 generations and samples were recorded every 1000 generations. The first 25% of the samples were discarded as burn-in, and the remaining samples were used to summarize the Bayesian posterior probabilities. ML analyses of the molecular dataset were conducted with IQ-TREE v2, and the node support values were assessed by bootstrap resampling with 1000 replicates. Finally, FigTree v1.3.1 [34] was used to visualize the phylogenetic tree.

MCMCTree from the PAML 4.9 package was used to estimate divergence times in *Tliponius* with the correlated rates model (clock = 3) based on the ML tree of 32PCGs [35]. The only fossil of *Homoeocerus*, *Homoeocerus attenuatus* (17–21 Ma), was used in the calculation before the clade of *H. marginiventris* because the fossil record is similar to the characters of this current species [36]. The main parameters during operation are as follows: model = 0, rgene_gamma = 2, 20. After a 20,000 burn-in, the MCMCTree program was run for 1,000,000 MCMC steps and sampled every 100. The robustness of the MCMCTree results was checked by comparing the consistency of at least two independent runs, with all parameters at least 200 for the effective sample sizes.

### 2.5. Molecular Species Delimitation

Two different mitochondrial datasets (32COI and 32PCGs) were used by four methods for species delimitation. (1) Automatic Barcode Gap Discovery (ABGD) is an automatic procedure that uses pairwise genetic distance to determine the barcode gap and sorts sequences into hypothetical species. ABGD analysis was conducted (http://galaxy.itaxotoolsweb.org/?tool_id=ABGD&version=latest, accessed on 14 April 2024) with the default settings and the Kimura 2-P (K80) distance model [37]. (2) Assemble Species by Automatic Partitioning (ASAP) analysis was conducted online (http://galaxy.itaxotoolsweb.org/?tool_id=ASAP&version=latest, accessed on 14 April 2024) using the Kimura (K80) ts/tv 2.0 substitution model for distance calculations [38]. (3) Bayesian Poisson tree process (bPTP) is an updated version of the original maximum likelihood PTP, and it adds Bayesian support values to the input tree to delimit species. bPTP analysis used the output from IQ-TREE v2 as input to conduct delimitation (https://species.h-its.org/ptp/, accessed on 15 April 2024) [39]. (4) Generalized Mixed Yule Coalescent (GMYC) model integrates both Yule and coalescent models to identify species boundaries in an ultrametric tree [40]. An ultrametric tree was eventually generated by BEAST2 [41]. We used the Yule model tree prior and a strict clock, running 10 million generations and sampling every 1000 generations. Finally, GMYC delimitation was run with the ultrametric tree as input and a single-threshold method on the web server (https://species.h-its.org/gmyc/, accessed on 15 April 2024).

Genetic distances between individuals and species groups (grouped following the clusters from species delimitation results) were examined using the COI sequence via uncorrected pairwise genetic distance as implemented in MEGA X [22].

Population genetic analysis was conducted to characterize population genetic structure and population genetic differentiation based on SNP datasets (28SNP and 28uSNP). Population genetic structure was estimated using two methods: Bayesian clustering and discriminant analysis of principal components (DAPC). Bayesian clustering was conducted in STRUCTURE 2.3.4 [42]. We ran STRUCTURE 10 times for each K-value from 2 to 10. Each run was performed for 120,000 MCMC generations with the first 20,000 generations discarded as burn-in. STRUCTURE HARVESTER (https://web.archive.org/web/*/http://taylor0.biology.ucla.edu/structureHarvester/, accessed on 2 January 2024) was used to determine the optimal K-value; 10 runs were combined into one output in CLUMPP v.1.1.2 [43], and the results were displayed graphically in DISTRUCT [44]. DAPC analysis was conducted in the R package *adegenet*. We used the grouping results based on Bayesian clustering analysis as priors and the “xvalDapc() command” to determine the optimal number of PCs. Phylogenetic networks were built in SplitsTree4 v.4.14.5 using the neighbor-net method [45].

## 3. Results

### 3.1. Information Contained in the Dataset and the Phylogenetic Partitioning Model

For the mitochondrial dataset, we obtained a matrix of 13 PCGs (32PCGs) with a length of 11,043 bp and a matrix of COI (32COI) of 1533 bp. The best-fit nucleotide substitution models (Table S2) were used for phylogenetic inference and divergence time estimation.

For the SNP dataset, we acquired a SNP dataset with a total of 35,486 SNPs (28SNP) and an uSNP dataset with 1115 unlinked SNPs (28uSNP).

### 3.2. Molecular Species Delimitation

#### 3.2.1. Species Delimitation Based on Mitochondrial Data

**Figure 2:**
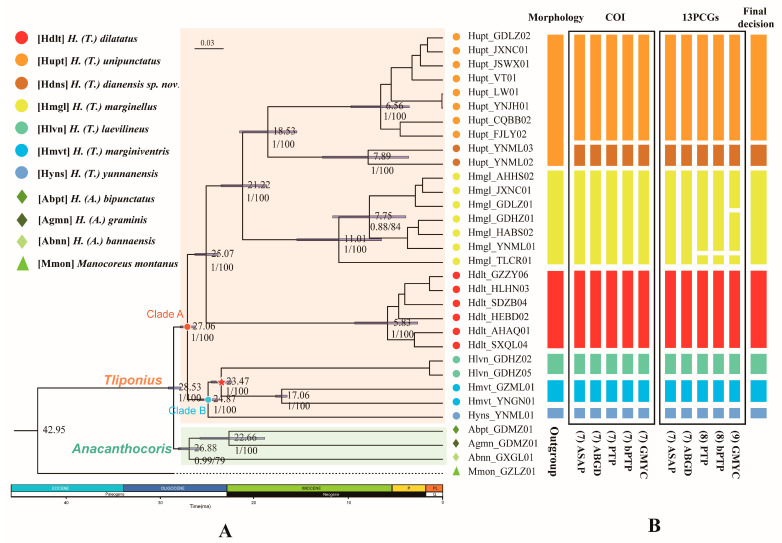
Phylogenetic tree and species delimitation hypotheses for *Tliponius*. (**A**) Phylogenetic inference and divergence time estimation based on the PCG123 dataset. Median age estimates are presented on nodes; the blue bars indicate the 95% highest probability density intervals. The numbers under the nodes are maximum likelihood bootstrap values (ML)/Bayesian posterior probabilities (BI). The red pentagram of the node represents the fossil calibration point. P, Pliocene; PL, Pleistocene. (**B**) The result of the species delimitation analyses. The columns are the results of initial morphological identification, five delimitation methods based on molecular data (ABGD, ASAP, PTP, bPTP, and GMYC) based on the COI gene and the concatenated 13 PCGs and the final decision. The putative species (MOTUs) inferred by the molecular data are shown in different colors.

The various methods applied to two mitochondrial datasets (32COI and 32PCGs) resulted in 7, 8, or 9 molecular operational taxonomic units (MOTUs) for the species of *Tliponius* (Figure 2B).

The different divisions of MOTUs mainly focus on the division of Yunnan samples of *H. unipunctatus* and the whole sample of *H. marginellus*. The other five species were divided consistently in terms of their morphological identity.

First, 7-MOTU classification distinguished two Hupt_YNML samples from the other *H. unipunctatus* individuals, and simplified them into a new MOTU component. Two distance-based methods (ASAP and ABGD) supported this division with COI and PCGs datasets. Three tree-based method (GMYC, PTP and bPTP) analyses using the COI dataset are also consistent with the above results. Second, the main difference between 8-MOTUs and 7-MOTUs is that the PTP and bPTP analyses regard the Hmgl_TLCR01 sample in *H. marginellus* as an independent MOTU in the PCGs dataset. Finally, the division results of the 9-MOTUs further divided the other samples of *H. marginellus* into two separate MOTUs, which was supported by GMYC analysis of the 13 PCGs dataset.

Genetic distance analysis based on COI revealed that the genetic divergence between the two Hupt_YNML samples was only 1.31%. In contrast, the genetic distance between these two samples and other *H. unipunctatus* samples exceeded 10.37%, and the distance to another Yunnan-distributed *H. unipunctatus* sample (Hupt_YNJH01) was greater than 10.44%. In comparison, the genetic variation among *H. unipunctatus* samples within the same MOTU ranged from 0.00% to 1.38%. For other species, the widespread *H. marginellus* showed a relatively wide range of intraspecific genetic variation (0.65–3.59%), with the maximum genetic distance observed in sample Hmgl_TLCR01, which was separately grouped in the tree-based method based on the PCGs dataset. Another widespread species, *H. dilatatus*, presented a narrow range of intraspecific genetic variation (0.98–1.58%). Additionally, the intraspecific genetic variation in *H. laevilineus* and *H. marginiventris* were 0.33% and 0.72%, respectively (Table S10). In accordance with the majority of the species delimitation results, the minimum interspecific genetic distance was 10.55% (Table S11), with the minimum genetic distance between samples at 10.51%.

#### 3.2.2. Population Genetics Analysis Based on SNP Data

**Figure 3:**
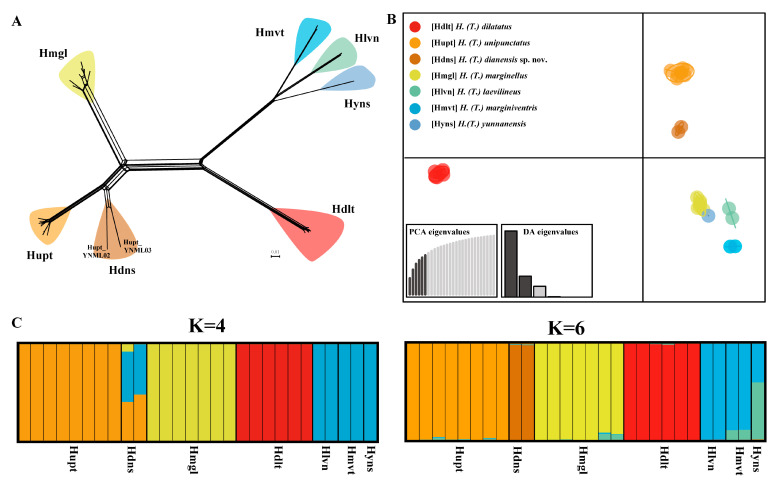
Population genetic structure of *Tliponius* based on the 28SNP dataset. (**A**) Phylogenetic networks constructed using the neighbor-net method. (**B**) Discriminant analysis of principal components (DAPC) results. (**C**) STRUCTURE results when K = 4 and K = 6 (Different colors in STRUCTURE denote distinct ancestral genetic clusters).

The phylogenetic networks based on the SNP dataset divided *Tliponius* into seven clades (Figure 3A). For the *H. unipunctatus* samples, two YNML samples formed a separate branch distinct from those of the other in *H. unipunctatus* samples, which is consistent with the species delimitation results based on the mitochondrial genome. However, the samples from *H. marginellus* presented a short split in their own branch without an independent split in the mitochondrial delimitation. The other four species were well clustered into separate groups. *H. laevilineus*, *H. marginiventris* and *H. yunnanensis* are relatively closely-related but distinctly independent lineages.

The DAPC analysis also revealed that the species in this subgenus were divided into seven groups (Figure 3B). *H. unipunctatus* was divided into two groups, and the other species formed single groups respectively, which is consistent with the 7-MOTUS delimitation results based on the mitochondrial genome.

The optimal value of K in the STRUCTURE analysis was 4, but when K = 6, the results clearly revealed the genetic components of different species (Figure 3C). When K = 4, *Homoeocerus dilatatus*, *H. unipunctatus* (except for the Hupt_YNML samples marked Hdns) and *H. marginellus* have independent genetic components, whereas the three species *H. laevilineus*, *H. marginiventris* and *H. yunnanensis* have common independent genetic components. For the Hupt_YNML samples, a mixture of three genetic components was presented, which was significantly different from the remaining *H. unipunctatus* samples. When K = 6, *Homoeocerus dilatatus*, *H. marginellus*, *H. unipunctatus* (except for the Hupt_YNML sample) and Hupt_YNML all have their own independent components. Furthermore, although *H. laevilineus*, *H. marginiventris* and *H. yunnanensis* contain common components, these three species can be effectively distinguished by the different contents of another component.

### 3.3. Validation by Morphological Re-Examination

Based on the distinct divergence revealed by species delimitation analyses and molecular data, we re-examined the morphological characters of the specimens. Specimens identified as *H. marginellus* were morphologically consistent across populations. In contrast, the YNML specimens, which were initially assigned to *H. unipunctatus* based on external morphology, exhibit darker coloration on antennal segments II and III and deeper abdominal punctures than other specimens of *H. unipunctatus*. Further dissection of the YNML specimens revealed subtle but consistent differences in the genital segments of both sexes compared to *H. unipunctatus* (see Section 5, “Taxonomy”). These morphological distinctions, together with molecular evidence, support the recognition of the Hupt_YNML lineage as an independent clade that warrants the erection of a new species, *H.* (*T.*) *dianensis* Liang, Li & Bu sp. nov.

### 3.4. Phylogenesis and Divergence Time

We constructed phylogenetic trees for seven possessive species based on a mitochondrial dataset (32PCGs) and nuclear gene dataset (32SNPs) (Figure 2A). Based on the ML and BI methods, species of the *Tliponius* subgenus are able to form a stable and independent monophyletic state. The seven species in *Tliponius* presented a relatively stable and consistent topology: Clade A contained (*H. dilatatus* + (*H. marginellus* + (*H. unipunctatus + H. dianensis* sp. nov.))), Clade B contained (*H. yunnanensis* + (*H. laevilineus* + *H. marginiventris*)).

The divergence time estimates indicate that the subgenera *Tliponius* and *Anacanthocoris* diverged in the Oligocene ca. 28.53 Ma (95% HPD: 27.77–29.08 Ma). The diversification of the two clades in *Tliponius* mainly occurred at approximately 27.06 Ma (95% HPD: 26.21–27.83 Ma). In Clade A, *H. dilatatus* separated at approximately 25.07 Ma (95% HPD: 23.80–26.28 Ma), *H. marginellus* separated at approximately 21.22 Ma (95% HPD: 18.66–23.5 Ma), and *H. unipunctatus and H. dianensis* sp. nov. separated at 18.53 Ma (95% HPD: 15.45–21.55 Ma). In Clade B, *H. yunnanensis* first separated at approximately 24.87 Ma (95% HPD: 23.83–25.86 Ma), and then *H. laevilineus and H. marginiventris* separated at 23.47 Ma (95% HPD: 22.33–24.5 Ma) (Figure 2A).

### 3.5. Comparison of the Mitogenome of Tliponius

**Figure 4:**
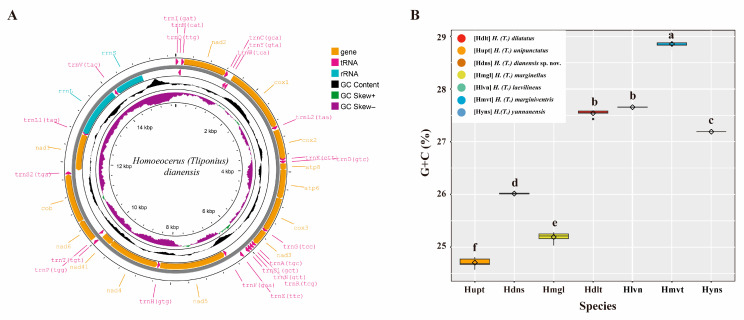
(**A**) Mitogenome map of the new species, *H. dianensis* sp. nov. The direction of gene transcription is indicated by the arrows on the strands. PCGs and rRNAs are represented by normative abbreviations, while tRNAs are indicated by single−letter abbreviations. (**B**) GC content in 13 PCGs of each species in *Tliponius*. Different letters are significantly different at *p* < 0.05 (ANOVA).

We obtained 31 new mitogenomes of *Homoeocerus* species, 28 belonging to the subgenus *Tliponius* and 3 belonging to the subgenus *Anacanthocoris*. There are 28 mitogenomes ranging in length from 14,542 to 14,676 bp (without the control region). All the mitogenomes were extremely conserved and included 37 canonical mitochondrial genes without rearrangement events (Figure 4A). The whole mitogenome exhibited the typical A + T biased composition of insects. The A + T content of the mitochondrial genome ranged from 71.95% to 76.02%. All the obtained mitogenomes had presented positive AT-skew and negative GC-skew. We obtained a matrix of 13 PCGs with a length of 11,043 bp and a matrix of COI of 1533 bp. The G + C content of the 13 PCGs ranged from 24.56% to 28.89% (Table S13). Notably, the nucleotide compositions of the PCGs clearly differed among *Homoeocerus* species (*F* = 1650, *p* < 0.001), but the same species presented similar GC contents. The average content of *H. dilatatus* was 27.54%, that of *H. marginellus* was 25.19%, that of *H. laevilineus* was 27.66%, that of *H. marginiventris* was 28.85%, and that of *H. yunnanensis* was 27.19%, and the GC content of *H. dianensis* sp. nov. in 26.01% was significantly different from that of *H. unipunctatus* in 24.70% (*p* < 0.001) (Figure 4B).

## 4. Discussion

Although some slight morphological differences between Hupt_YNML samples and other *H. unipunctatus* samples were observed (e.g., the shade of the abdominal punctures and the intensity of the antennal color), they were not initially solid enough to be direct evidence for delimiting species. Intraspecific morphological variation is common in many species, especially widely distributed ones, making further molecular evidence necessary to confirm species validity.

All species delimitation analyses based on the mitochondrial datasets supported Hupt_YNML samples as an independent MOTU. Seven species were well revealed by all distance-based species delimitation methods and all tree-based methods based on COI data. Moreover, the genetic distance results based on the COI for the seven species revealed that the maximum value of genetic variation within the species was 3.59%, and the interspecific genetic distances within the genus mostly exceeded 10%. The genetic distance between *H. dianensis* sp. nov. and *H. unipunctatus* was 10.55%, which was far greater than the maximum intraspecific genetic distance, strongly supporting the species status of the new taxon (Table S10).

In the delimitation of the widespread species *H. marginellus*, tree-based methods (PTP, bPTP, and GMYC) based on 13PCGs have divided it into 2 or 3 MOTUs. However, considering the morphological consistency and the clearly singular grouping results of SNP data, these samples should all be regarded as the same species. Tree-based methods have exhibited over-splitting in many previous studies [46]. In this study, the multiple lineages of *H. marginellus* were characterized by geographical aggregation. Therefore, such a division may reflect the high intraspecific genetic differentiation and potential phylogeographic pattern structure among populations within the species.

In population genetics analyses based on nuclear SNP data, Hupt_YNML samples also presented substantial genetic differentiation from *H. unipunctatus*, particularly in terms of long branch lengths in the NN tree (Figure 3A). In the STRUCTURE analysis, genetic clustering differences between the two groups were already evident at K = 4 and at K = 6. Both formed independent and distinct genetic clusters, strongly supporting their status as separate species (Figure 3C). For the other two widespread species, *H. dilatatus* and *H. marginellus*, intraspecific differentiation was low, which is consistent with the mitochondrial species delimitation results. The resolution for *H. laevilineus*, *H. marginiventris*, and *H. yunnanensis* was notably poor based on nuclear SNP data, primarily due to the final ddRAD data matrix containing only five samples for these species, a high percentage of missing data (N), and few informative loci collectively reducing the precision of interspecific resolution [47].

Considering the delimitation results of both the mitochondrial genomes and the nuclear SNPs, we accepted the seven-species delimitation result and named the Hupt_YNML samples as *H. dianensis* sp. nov., which possesses specific morphological characters differing from *H. unipunctatus* (for detailed morphological descriptions, see Section 5, Taxonomy). Moreover, *H. unipunctatus* itself is readily distinguishable from the other 43 known species of the subgenus *Tliponius*, and *H. dianensis* sp. nov. likewise differs from all other described species within this subgenus, confirming its status as a distinct species rather than a variant of any known taxa. Thus, *H. dianensis* sp. nov. represents a cryptic species that was previously overlooked within the broad species concept of *H. unipunctatus*.

For widely distributed taxa, accurate species delimitation should be based on extensive sampling, particularly for marginal populations distributed in areas with special geological environments. In our study, the new species was discovered in Yunnan Province, which is located at the edge of the Hengduan Mountains. This region is considered a hotspot for species diversification because of its unique geological history and complex topographical patterns. Its complex terrains (such as the Yunnan–Guizhou Plateau and Honghe Valley) and diverse climate types make it a primary hotspot for discovering cryptic species [48,49,50,51]. The divergence time estimates suggested that *H. dianensis* sp. nov. diverged from *H. unipunctatus* approximately 18.53 Ma, during the Early Miocene. This period coincides with significant geological events in southwestern China, particularly the accelerated uplift of the eastern Tibetan Plateau and the formation of the Hengduan Mountains [52,53]. Thus, widespread species may undergo local adaptation in Yunnan’s heterogeneous habitats, gradually accumulating genetic differentiation [54,55,56,57]. Therefore, the discovery of *H. dianensis* sp. nov. in this region not only underscores the importance of marginal population sampling in species delimitation but also provides a potential case for geological complexity driving the formation of cryptic species in biodiversity hotspots.

Owing to the limited number of samples used in this study, while we were able to confirm the validity of the new species, we could not fully resolve the phylogenetic relationships among closely related species. Future research should strive to incorporate more comprehensive species sampling to clarify the phylogenetic relationships within the subgenus and shed light on its evolutionary history.

## 5. Taxonomy

**Key to the species of the subgenus *Homoeocerus* (*Tliponius*) in China**

1. Corium with a small black spot in the center ………………………………………………………………………………………………………………………… 2

- Corium without a spot in the center …………………………………………………………………………………………………………………………………… 6

2. Abdomen distinctly expanded laterally in the middle and posterior parts; antennal segments II and III distinctly triangular and significantly flattened; no distinct longitudinal black stripes behind the compound eyes on both sides of the head …………………………………*H.* (*T.*) *dilatatus* Horvath, 1879

- Abdomen slightly expanded laterally or not expanded in the middle and posterior parts; antennal segments II and III cylindrical, or triangular but not flattened …………………………………………………………………………………………………………………………………………………………………… 3

3. Antennal segment I slightly triangular, with small black granules; connexivum with dense small black spots; setae on the 3rd and 4th abdominal ventral segments without black margins ……………………………………………………………………………………………………………………………… 4

- Antennal segment I not triangular, granules not black; connexivum light-colored, without black punctures; setae on the 3rd and 4th abdominal ventral segments with distinct black margins …………………………………………………………………………………………………………………………………… 5

4. Antennal segments II and III concolorous with segment I, yellowish-brown; black spots on the abdominal ventral surface small and sparse; female with extending posterior margin of abdominal sternite VII forming curved inward angles; male with basal angle of paramere obtuse …………… *H.* (*T.*) *unipunctatus* (Thunberg, 1783)

- Antennal segments II and III darker and reddish compared to segment I, segment II blackish; black spots on the abdominal ventral surface larger and denser; female with extending posterior margin of abdominal sternite VII forming angles without distinct inward curvature; male with basal angle of paramere sharp ……………………………………………………………………………………………………………… *H.* (*T.*) *dianensis* Liang, Li & Bu sp. nov.

5. Antennae longer, segment II equal to or slightly shorter than the width of the pronotum …………………… *H.* (*T.*) *marginellus* (Herrich-Schaeffer, 1840)

- Antennae shorter, segment II about 3/5 the width of the pronotum ……………………………………………………………… *H.* (*T.*) *pallidulus* Blöte, 1936

6. Abdominal spiracles without black margins ………………………………………………………………………………………………………………………… 7

- Abdominal spiracles with black margins ……………………………………………………………………………………………………………………………… 8

7. Head and pronotum with black margins; antennal segments II and III with black tips; lateral angles of the pronotum not prominent ……………. *H.* (*T.*) *sinicus* Walker, 1871

- Head and pronotum without black margins; antennal segments II and III not black-tipped; lateral angles of the pronotum prominent, approximately right-angled and upturned ………………………………………………………………………………………………………………… *H.* (*T.*) *insignis* Hsiao, 1963

8. Connexivum with 2 or 3 black spots on each segment; a narrow brown longitudinal band in the middle of the abdominal ventral surface (Plate IX c) ………………………………………………………………………………………………………………………………………… *H.* (*T.*) *marginiventris* Dohrn, 1860

- Connexivum without black spots; no brown longitudinal band in the middle of the abdominal ventral surface ……………………………………………………………………………………………………………………………………………………………………………… 9

9. Head and abdominal ventral surface with dense black punctures; rostrum with the II segment longer than the III segment ……………… *H.* (*T.*) *yunnanensis* Hsiao, 1962

- Head and abdominal ventral surface relatively smooth, with dense black punctures; rostrum with the II segment shorter than the III segment ……………………………………………………………………………………………………………………………………………………………………………… 10

10. Body smaller and narrower; pro-, meso-, and metathoracic sides with 1, 2, and 1 distinct black spots respectively …………………………… *H.* (*T.*) *laevilineus* Stål, 1873

- Body slightly larger and wider; only meso- and metathoracic sides with 1 distinct black spot each ………………………………………………………… 11

11. Posterior margin of the head black; a complete smooth stripe from the center of the pronotum to the scutellum ………………………… *H.* (*T.*) *shokaensis* Matsumura, 1913

- Posterior margin of the head concolorous; a short longitudinal line at the anterior center of the pronotum, only discernible in the front …………………………………………………………………………………………………………………..…………………………… *H.* (*T.*) *cingalensis* Stål, 1860

**Figure 5:**
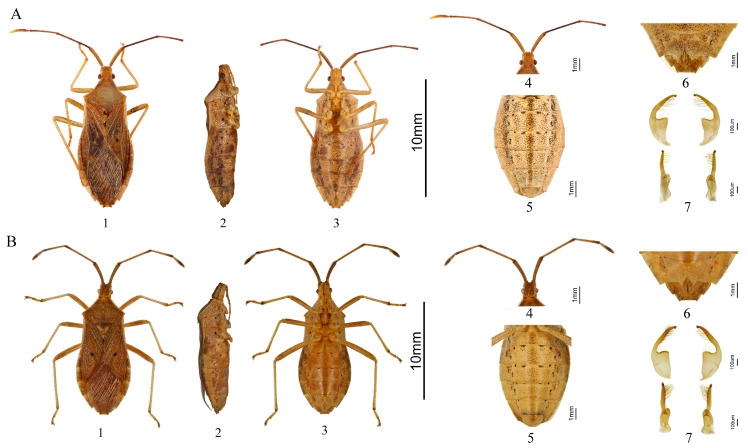
Morphological comparison between *H. dianensis* sp. nov. and *H. unipunctatus*. (**A**) *H. dianensis* sp. nov. (**B**) *H. unipunctatus*. 1. Dorsal view; 2. Side view; 3. Ventral view; 4. Head; 5. Abdomen; 6. The seventh abdominal segment of the female insect; 7. Paramere, four different views.

***Homoeocerus*** **(*Tliponius*) *dianensis* Liang, Li & Bu sp. nov. (Figure 5A)**

urn:lsid:zoobank.org:act:162C8032-FF9D-4673-8463-55735B97A599

**Diagnosis:** This new species (Figure 5A) closely resembles *H. (Tliponius) unipunctatus* (Thunberg, 1783) (Figure 5B), but can be distinguished from *H. unipunctatus* and all other known species of *Homoeocerus* (*Tliponius*) by the following combination of characters: antennal segments II–III dark, segment II nearly black (vs. yellowish-brown in *H. unipunctatus*) (Figure 5(A4,B4)); black spots on the ventral abdomen darker with thicker, more distinct edges (vs. paler with thinner edges) (Figure 5(A5,B5)); posterior margin of abdominal sternite VII extending laterally to form acute angles without distinct inward curvature (vs. forming curved angles directed inward) (Figure 5(A6,B6)); basal angle of paramere sharp (vs. obtuse) (Figure 5(A7,B7)).

**Description:** Body oblong-oval, densely punctate with light black dots. Dorsum of body yellowish-brown. Ventral of head, ventral of thorax, legs, and ventral of abdomen light yellow. Eyes dark brown, ocelli light red.

The length of the head longer than its width; posterior half with longitudinal groove. Dorsal surface of head with small light black protrusion. Antennae cylindrical. Antennal segments I swollen, thickest and yellowish-brown. Antennal segments II–III slender and dark. Antennal segments IV fusiform, with base yellowish-brown and apex light-yellow. Antennal segments I–III speckled with small dark black granular projection. Rostrum between procoxae and mesocoxae, with dark apex; length of 1–4th segments (mm): 0.76, 0.76, 0.78, 1.24.

Pronotum evenly punctulate in the anterior half, anterior lateral margin light yellow, lateral angles relatively sharp but not prominent. The inner side of the center of the mesosternum and the center of the metasternum near the posterior margin with a large black spot respectively. Scutellum with black punctations. Femora robust with small black granular projection, tibiae and tarsi slender, claws with dark black apex. Corium of the fore-wings with a small black spot in the center. Hemelytra not reach the apex of abdomen, lateral connexivum mostly exposed.

Abdominal sternite III–IV with three large black spots on the outer side of the midline. Abdominal sternite V–VI with two large black spots on the outer side of the midline. Lateral connexivum dorsally densely covered with black punctures. Abdominal venter densely covered with large black punctures. Abdominal spiracles colorless.

Female: Posterior margin of abdominal sternite VII extending and expanding along both sides of the median suture, forming acute angles (Figure 5(A6)).

Male: The base of paramere broad and nearly triangular, the angle at the intersection of the upper terminal margin and the inner margin sharp. The apical half of the male paramere club-shaped, gradually narrowing from the base to the apex, tip forming short and blunt beak-like (Figure 5(A7)).

**Measurements (mm):**

♀: body length 12.10–12.33; head length 0.84–0.92; head width 0.92–0.96; lengths of antennal segments I-IV 1.85–1.96, 3.06–3.21, 2.06–2.20, and 1.55–1.69, respectively; pronotum length 2.22–2.47; pronotum width 3.65–3.82; scutellum length 1.37–1.49; basal width of scutellum 1.54–1.67; maximum abdominal width 4.70–4.90.

♂: body length 10.54–10.88; head length 0.84–0.89; head width 0.87–0.94; antennal segments I-IV lengths 1.73–1.79, 2.79–2.84, 2.03–2.19, and 1.49–1.52; pronotum length 1.70–1.81; pronotum width 3.26–3.35; scutellum length 1.40–1.43; basal width of scutellum 1.49–1.57; maximum abdominal width 4.44–4.58.

**Molecular data: COI** PV988320 (GenBank).

**Etymology:** The species is named according to the current collection information and sample records. As the species is discovered in the Yunnan Province, the specific epithet Dian is derived from the Chinese abbreviation of Yunnan.

**Distribution:** China (Yunnan).

**Type material:**

Holotype: ♀, China: Yunnan: Mengla County, Shangyong Town, 20.ix.1979 (Zuopei Ling leg.) (pinned, NKUM). Paratypes: China: Yunnan Province: 1♂, same data as the holotype (pinned); 1♂, 1♀, Dehong Prefecture, Mang City, 24.42 °N, 98.59 °E, alt: 1300 m, 1.viii.2006 (Jimon Hua leg); 1♂, Jinghong City, Menglong Town, Manan Village, 21.90 °N, 101.26 °E, alt: 495 m, 6.vii.2018 (Shujing Wang leg.); 1♀, Jinghong City, Menglong Town, Mangsong Village, 21.49 °N, 100.50 °E, alt: 1543 m, 9.vii.2018 (Yujie Duan leg.); 2♂♂, Menghai County, Bulang Township, 21.62 °N, 100.40 °E, alt: 1188 m, 12.viii.2013, (Lizhi Cui & Xiaosheng Chen leg.); 1♂, Mengla County, 21.19 °N, 101.69 °E, alt: 879 m, 18.viii.2014 (Haiguang Zhang leg.); 1♀, Mengla County, Mengban Town, 21.74 °N, 101.63 °E, alt: 821 m, 22.viii.2014 (Penglei Guo leg.); 1♂, 1♀, Mengla County, Tropical Botanical Garden, rubber plantation plot 14–5, 21.90 °N, 101.27 °E, alt: 636 m, 1.viii.2012 (Guo Zheng leg.) (rest in ethanol, all in NKUM).

**Remarks:** In light of the morphological similarity between *H. dianensis* sp. nov. and *H. unipunctatus*, confirming the nomenclatural validity of the new species was necessary. Accordingly, we reviewed the taxonomic history of *H. unipunctatus* and its three junior synonyms: *H. chinensis*, *H. punctipennis*, and *H. distinctus*. The synonymy of *H. chinensis* and *H. punctipennis* is well supported [58,59]. *H. distinctus* was treated as a variety of *H. unipunctatus* by Hsiao (1962) for some specimens in Yunnan, China, shared the characters of the black antennae in segment II and III [22]. However, due to the absence of type material and the limited original description of *H. distinctus*, which only mentions black antennae and a small yellowish-gray terminal antennal segment with black punctures [21]. The original description did not specify which antennal segments were black. The other character described as “four black points at the base of the first and second ventral segments” (actually referring to the third and fourth abdominal sternites) is also present in *H. unipunctatus*, and thus cannot serve as a diagnostic feature for *H. distinctus*. Besides, no genital characters were described and the molecular data are lacking for *H. distinctus*. The best way to deal with this situation is to agree with the Hsiao (1962)’s treatment. We therefore describe the newly discovered lineage from Yunnan, China as a distinct species.

## 6. Conclusions

We employed an integrative taxonomic approach based on morphological characters and molecular data (COI, mitogenome, and ddRAD SNPs), combined with multiple species delimitation and population genetic methods, to reveal one new species (*Homoeocerus* (*Tliponius*) *dianensis* Liang, Li & Bu sp. nov.) from one originally widely distributed species. Based on our findings, extensive sampling of widespread species is highly important for the accuracy of species delimitation and the discovery of cryptic species. Based on the results of the current phylogenetic and divergence time analyses, the subgenus *Tliponius* is divided into two clades, with a divergence time of approximately 27.06 Ma between them. Clade A contained four species and presented as (*H. dilatatus* + (*H. marginellus* + (*H. unipunctatus + H. dianensis* sp. nov.))), Clade B contained three species and presented as (*H. yunnanensis* + (*H. laevilineus* + *H. marginiventris*). The divergence of the new species *H. dianensis* sp. nov. is estimated to have occurred approximately 18.53 Ma. Species delimitation plays a crucial role in identifying cryptic species within widespread taxa, thereby enhancing our understanding of biodiversity. Additionally, the use of a combination of methods such as coalescent-based species delimitation, population genetics analysis and morphometric analyses can increase the reliability of species delineation, particularly in similar taxonomic groups.

## Data Availability

All genetic dataset can be downloaded at the following link: https://doi.org/10.6084/m9.figshare.29312900.

## Supplementary Materials

The following supporting information can be downloaded at: https://www.mdpi.com/article/10.3390/insects16080797/s1. Table S1: Experimental sample information; Table S2: Best-fit models of sequence evolution and partitioning schemes selected by PartitionFinder2 for phylogenetic reconstructions; Table S3: Species delimitation results based on the COI dataset by ASAP; Table S4: Species delimitation results based on the COI dataset by ABGD; Table S5: Species delimitation results based on the COI dataset by GMYC; Table S6: Species delimitation results based on the COI dataset by PTP/bPTP; Table S7: Species delimitation results based on the 13PCGs dataset by ASAP; Table S8: Species delimitation results based on the 13PCGs dataset by ABGD; Table S9: Species delimitation results based on the 13PCGs dataset by GMYC; Table S10: Species delimitation results based on the 13PCGs dataset by PTP/bPTP; Table S11: The COI genetic distance of individuals; Table S12: The COI genetic distance of species group; Table S13: Composition and skewness in *Tliponius* mitogenome.
